# A Fusion-Based Machine Learning Approach for Autism Detection in Young Children Using Magnetoencephalography Signals

**DOI:** 10.1007/s10803-022-05767-w

**Published:** 2022-10-03

**Authors:** Kasturi Barik, Katsumi Watanabe, Joydeep Bhattacharya, Goutam Saha

**Affiliations:** 1 Department of Electronics and Electrical Communication Engineering, Indian Institute of Technology Kharagpur, Kharagpur, India; 2 Faculty of Science and Engineering, Waseda University, Tokyo, Japan; 3 Department of Psychology, Goldsmiths, University of London, London, UK

**Keywords:** Autism spectrum disorder, Brain oscillations, Preferred phase angle, MEG, Classification, Biomarker

## Abstract

In this study, we aimed to find biomarkers of autism in young children. We recorded magnetoencephalography (MEG) in thirty children (4–7 years) with autism and thirty age, gender-matched controls while they were watching cartoons. We focused on characterizing neural oscillations by amplitude (power spectral density, PSD) and phase (preferred phase angle, PPA). Machine learning based classifier showed a higher classification accuracy (88%) for PPA features than PSD features (82%). Further, by a novel fusion method combining PSD and PPA features, we achieved an average classification accuracy of 94% and 98% for feature-level and score-level fusion, respectively. These findings reveal discriminatory patterns of neural oscillations of autism in young children and provide novel insight into autism pathophysiology.

## Introduction

Autism spectrum disorder (ASD) is a complex neurodevelopmental disorder that influences the brain's information processing during infancy. Autism is characterized by disabilities in social association and communication, which generally exhibits repetitive behaviours and a restricted range of interests (Frith, 2008). Its prevalence rate in children is reported to vary from 0.23% in India (Rudra et al., 2017), 1.7% in the UK (Baio, 2014), to 2.5% in the USA (Xu et al., 2018). ASD influences individuals in various manners and can vary from mild to extreme (Amaral et al., 2017) and causes a significantly debilitating effect on the quality of life (Farley et al., 2009). There is no remedy for ASD, yet early identification followed by suitable intervention can reduce symptom severity, uphold improvement in behaviour and learning of an autistic child. Of note, predominant behavioural symptoms of ASD emerge later in the developmental phase, so there is a critical need for identifying early signs of ASD (Wolff et al., 2018).

This study aims to identify early neural markers of ASD in young children from their resting state neuromagnetic brain responses. A growing body of literature suggests that ASD is often associated with disruptions in the features of large scale brain oscillations (Billeci et al., 2013; Simon & Wallace, 2016), and this has been reported as the core feature of ASD pathophysiology in very young children (Gabard-Durnam et al., 2019). Electroencephalography (EEG) and Magnetoencephalography (MEG) are traditional neuroimaging techniques that record macroscopic brain activity with millisecond precision in a non-invasive fashion. Here, the MEG signal is preferred as it is reference-free. We recorded brain responses from young children between 4 and 7 years of age using a MEG device specially customized for children while they were watching cartoons of their choice. There were sixty children, equally divided into two groups: children with ASD and age-matched typically developing (TD) children.

One of the dominant theories of autism is based on the disturbance in the balance of excitation and inhibition (E/I) in key neural circuits (Rubenstein & Merzenich, 2003; Sohal & Rubenstein, 2019). It was found that altered excitation and inhibition were present in ASD due to increased or decreased high-frequency gamma-band activity based on context or task (Kessler et al., 2016). Literature report that the brain oscillatory changes in ASD are consistent with a disturbance in the balance of excitation and inhibition (Kessler et al., 2016; Port et al., 2019; Rubenstein & Merzenich, 2003; Simon & Wallace, 2016; Sohal & Rubenstein, 2019) as well as disruption in functional connectivity and altered thalamic function in multiple frequency bands of the oscillatory hierarchy (Simon & Wallace, 2016). The E/I imbalance can be surveyed non-invasively during a resting-state scan via patterns of neural oscillations that reflect the synchronous firing of large populations of neurons intervented by E/I interactions. In the literature of the last decade, phase-based features were investigated in the connectivity-based analysis (O'Reilly et al., 2017; Velazquez et al., 2009; Ye et al., 2014), but not in the spectral-domain analysis. Hence, we focus our analysis on phase-based features in the spectral domain for autism classification. We hypothesized that ASD might be reliably differentiated from normal brain function in young children by analyzing their ongoing brain oscillations power and phase in spectral-domain analysis. A study recently introduced phase angle clustering, a phase angle-based characteristic that quantifies the synchronisation of oscillatory activity within a conventional frequency spectrum (Barik et al., 2020). However, it is fundamentally different from phase-amplitude coupling (Port et al., 2019), a measure of functional connectivity. In the present study, the phase angle clustering property is the preferred phase angle (PPA). Since the angle of the average phase offset vector is the ‘preferred’ position of the phase angles of the frequency bins in a specific frequency band. In this study, along with the phase-based and power-based spectral features, we have combined these two complementary features in a machine learning classification framework for detecting ASD in a MEG data set.

Using pattern recognition and classification approach, this study presents an artificial neural network (ANN) based modelling for differentiating ASD children from TD children. As phase is independent of amplitude (Cohen, 2014), PPA based results are compared with frequently used power spectral density (PSD) based ones. Using the complementary characteristics of phase and power based features, a fusion based model is introduced in spectral-domain analysis to distinguish ASD from TD children by analyzing their ongoing large-scale neural responses. Findings show that the PPA feature yields better classification accuracy than PSD. Further, results highlight that dominant phase based features are mostly from theta band oscillations which correlates with the autistic symptomatology; however, the discriminating PSD features are mostly from high gamma band oscillations. The current study demonstrates a superior classification method by combining PSD and PPA based features in a fused system framework. In this paper, both feature-level fusion and score-level fusion are explored in autism children detection. In autism detection using MEG signals, this fusion-based method is a novel machine learning approach. This study investigates how different sets of children are misclassified in the individual model to understand the efficacy of this fusion-based technique of PSD and PPA features. Findings demonstrate that the multimodal fusion method effectively classifies ASD and TD children.

## Materials and Methods

### Participants

In this study, we focused on the age range of 4–7 years (47–86 months) as our primary goal is to identify early pathophysiological biomarkers of ASD in young children. There were two groups of children: (i) ASD and (ii) TD. The ASD group included 30 children (4 females) with autism spectrum disorder with a mean (± SD) age of 64.66 (± 10.12) months, and the TD group included 30 typically developing children (4 females) with a mean (± SD) age of 64.83 (± 10.51) months; the two groups did not significantly differ in age (two-tailed *t*-test, *p* > 0.95). The ASD children were diagnosed by an experienced psychologist and clinical psychiatrist using different screening tools; such as the Autism Diagnostic Observational Schedule, Generic (ADOS) (Rutter et al., 2003), the Diagnostic Interview for Social and Communication Disorders (DISCO) (Wing et al., 2002) and the DSM-IV (APA, 1994) criteria before taking the MEG data. Specifically, the ADOS cutoff and parent report on the social communication questionnaire are the popular screening tools with which the ASD was confirmed. The children in the TD group had no behaviour or language problems as reported by their parents. The written informed consents were obtained from the parents prior to the data acquisition. The Ethics Committee of Kanazawa University Hospital, Japan, approved this study protocol, and the experiment was conducted as per the World Declaration of Helsinki.

### Data Acquisition and Preprocessing

The MEG signals were acquired using a 151-channel superconducting quantum interference device (SQUID) whole head coaxial gradiometer MEG system (PQ1151R; Yokogawa/KIT, Kanazawa, Japan) that was customized for children in a magnetically shielded room (Daido Steel, Nagoya, Japan). The custom child-sized MEG system ensured the sensors were placed quickly and effectively to minimize head movement (Johnson et al., 2010). An experimenter was also present in the MEG room at the time of recording to ensure that the children remained relaxed to prevent movement throughout the recording. Resting MEG data were recorded from all children while they lay supine on the bed and viewed a silent cartoon of their own choice that was selected before the recording. There was no task involved, and the duration of the resting MEG data was 180 s. The sampling frequency was 1000 Hz and filtered online with a 200 Hz low-pass filter.

In this experiment, the preprocessing and analysis of MEG data were incorporated by MATLAB based toolbox, FieldTrip (Oostenveld et al., 2010), and by custom-made MATLAB scripts. The data were first visually inspected for large artefacts. Afterward, bad sensors that were either flat or noisy were detected and replaced by nearest-neighbour interpolation. Next, the MEG dataset was subsequently filtered between 1 and 100 Hz, and to reduce powerline noise, a notch filter was applied at 50 Hz. Finally, independent component analysis (ICA) was applied to remove eye-blink related artifacts.

### Data Analysis Overview

Given the role of large scale brain responses characterizing ASD, we divided the broadband MEG signal into six standard frequency bands (*fb*) (Donner & Siegel, 2011): delta-band (1–4 Hz), theta-band (4–8 Hz), alpha-band (8–13 Hz), beta-band (13–30 Hz), lower gamma-band (30–50 Hz) and higher gamma-band (50–100 Hz). Our primary focus was classifying ASD children from TD children using resting-state MEG signals. Towards this, we adopted a machine learning framework (Barik et al., 2019a, 2019b; Barik et al., 2019a, 2019b); machine learning based approaches are useful in diagnostic and intervention research in clinical neuroimaging (Iniesta et al., 2016; Vieora et al., 2017), including ASD (Hyde et al., 2019). Furthermore, experts have a certain degree of subjectivity (supported by rigorous statistical analysis) in creating the diagnostic instruments (i.e., ADOS, DISCO). Hence, it is reasonable to believe that objective machine learning methods may provide more reliable performance and increased efficiency by reducing redundancy within an instrument. The supervised machine learning framework consists of the following main blocks—feature extraction, feature selection and modelling which leads to the classification of two different classes, TD and ASD. We followed a nonliear artificial neural network (ANN) based modelling scheme (Bishop, 1995; Haykin & Network, 2004) to learn the underlying relationship among the selected fetaures of a particular class to separate it from the other class.

### Feature Extraction

Frequency domain analysis is the most common and familiar analysis for characterizing neural oscillations of a signal. Fourier Transform (FT) is the keystone of this frequency domain analysis. FT decomposes a time-domain signal into its constituent frequencies with different amplitude and phase angles (Oppenheim, 1999). It was found that phase angles are independent of magnitude (Cohen, 2014). We investigated the power spectral density (PSD) and phase-based features to compare classification performance. In this paper, we explained a frequency band-wise preferred phase angle (PPA) as a novel feature in MEG signal processing. The power spectral density of a continuous-time and finite power signal estimates the power of each of the constituent frequency components. For each channel of the MEG, the PSD is estimated using Welch’s method (Welch, 1967), where the whole time-series (180 s) is divided into equal 8 segments with 50% overlap. Hence, the length of each of the 8 segments is 40 s. Then, each segment is windowed with a Hamming window, and its magnitude squared FT is computed. The PSD estimate for each channel is obtained by averaging the periodogram estimates across segments, as shown below:

1 $$S_{xx} \left( f \right) = \frac{1}{N}\mathop \sum \limits_{n = 1}^{N} \left| {X_{n} \left( f \right)} \right|^{2} ; \;f\; = \;1,\;2, \ldots ,100\;{\text{and}}\;N\; = \;8$$

The frequency domain representation, $X_{n} \left( f \right)$ is the FTof time signal $x_{n} \left( t \right)$. After getting all the PSD over 1 to 100 Hz in steps of 1 Hz frequency components, we averaged the power spectrum over the six frequency bands as shown in the following equation.

2 $${\mathbf{PSD}}_{{{\mathbf{fb}}}} \; = \;10\log_{10} \left( {\frac{1}{{N_{fb} }}\mathop \sum \limits_{{f = fb_{\min } }}^{{fb_{\max } }} S_{xx} \left( f \right)} \right)$$

where *fb* represents each frequency band, and *N*_*fb*_ represents the number of components in each *fb*. For example, in case of alpha band, *fb*_*min*_ = 8, and *fb*_*max*_ = 13, so *N*_*fb*_ = 6, as our frequency resolution was 1 Hz. The frequency band-wise power spectral density estimates are denoted as *PSD*_*fb*_.

**Fig. 1:**
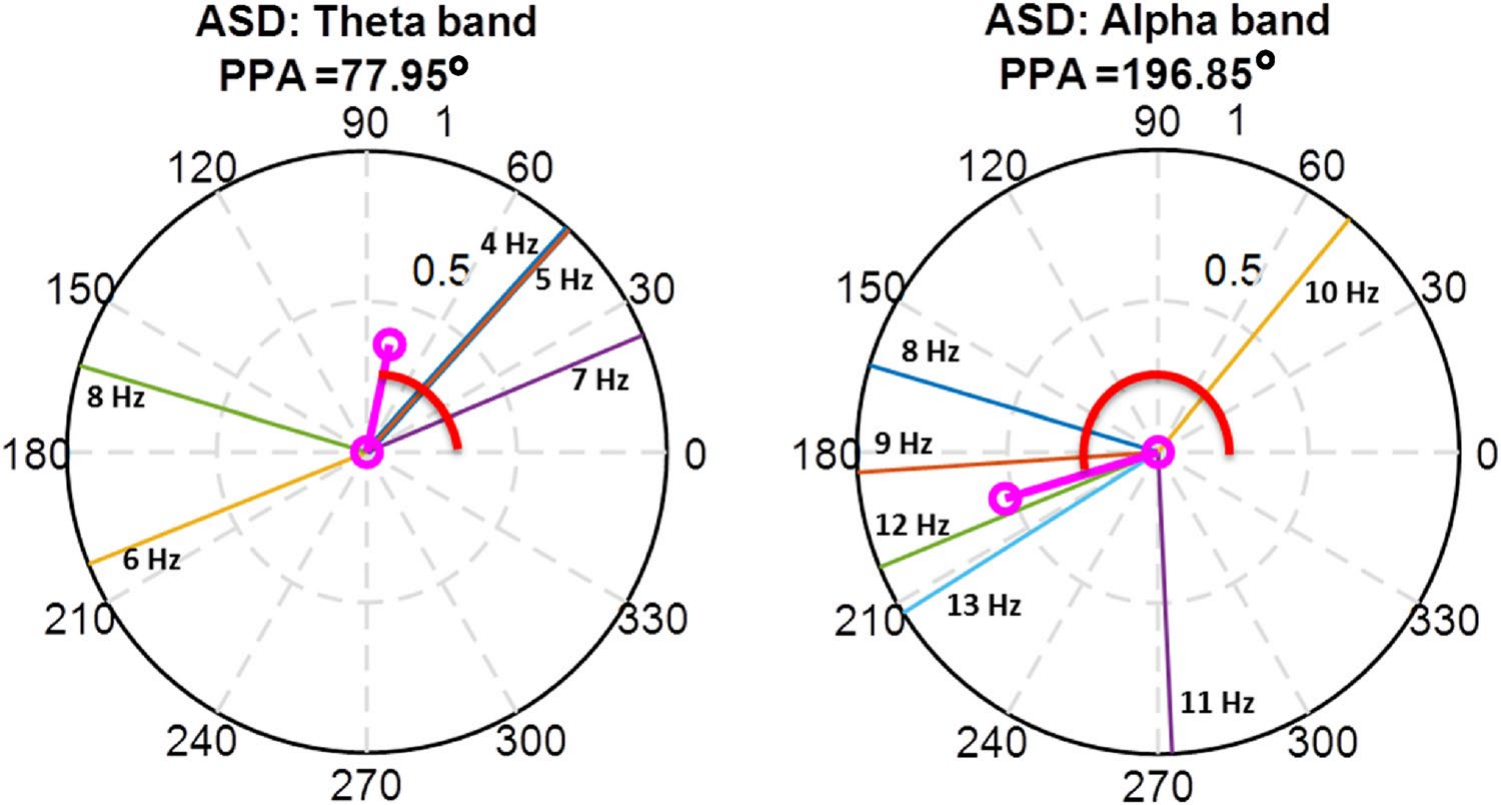
Presentation of PPA of theta band and alpha band (PPA_theta_ and PPA_alpha_) in the polar space (indicated by red color). The bold magenta line indicates the average phase consistency and the average phase angle represents PPA feature indicated by bold red color. In the first polar plot (left side) of theta band representation, the phase angles of 4 to 8 Hz frequency bins are represented by blue, orange, yellow, violet and green lines respectively. A similar color convention is followed in the right side polar plot for the alpha band with frequency bins ranging from 8 to 13 Hz in increasing order

A frequency band-wise phase angle-based feature named phase angle clustering was initially developed in a previous study (Barik et al., 2020). In this study, we have analyzed this phase-based feature in detail. After the computation of Fast Fourier Transform (FFT), we get phase angles (*θ*) at each frequency component (*f*) from 1 to 100 Hz in steps of 1 Hz for each channel (Oppenheim, 1999). The phase angle is calculated using simple Euler's formula $e^{{i\theta_{\left( f \right)} }}$ (Strang, 1991). Phase angles are commonly represented in polar space, ranging from 0 to 2*π*. These can be illustrated as vectors on a unit circle. In the polar space, the length of the average phase angle vector is known as phase consistency (Cohen, 2014), and the angle of this resultant phase vector is termed as preferred phase angle (PPA). For each MEG channel, in a given frequency band ($fb$), *PPA*_*fb*_ is calculated as:

3 $${\mathbf{PPA}}_{{{\mathbf{fb}}}} = \measuredangle \left( {\frac{1}{{N_{fb} }}\mathop \sum \limits_{{f = fb_{min} }}^{{fb_{max} }} exp^{{i\theta_{\left( f \right)} }} } \right)$$

where all the notations are similar to the first equation. Here, Fig. 1 is shown for a detailed illustration of **PPA**_**fb**_. Figure 1 represents the PPA of theta band (*PPA*_*theta*_), where each unit length phase angles depict 4 Hz, 5 Hz,…, 8 Hz frequency components. These phase angles of different frequency bins within a frequency band are represented by different colors (line of blue, orange, yellow, violet, green, cyan). The average phase angle represents PPA clustered of theta band frequencies (indicated by red color), and the average phase consistency (indicated by magenta color) is the length of the average phase angle vector in the polar space. Similarly, it is found for the alpha band in the right side from 8 to 13 Hz with the gap of 1 Hz frequency components. All the ASD subjects’ *PPA*_*theta*_ and *PPA*_*alpha*_ are shown in Fig. 1 from a randomly chosen channel (sensor: AG102). Here, in the polar space, *PPA*_*theta*_ value is 77.95°, and *PPA*_*alpha*_ value is 196.85°.

For comparison of classification performance, we computed commonly used power-based along with phase-based spectral domain features. In addition, *PSD*_*fb*_ and *PPA*_*fb*_ features are computed across all MEG channels. Hence, for both features (*PSD*_*fb*_ and *PPA*_*fb*_), the dimension for each participant is 906 [channel (151) × frequency-band (6)] for the overall brain, whereas, for each hemispheric features, the dimension is 420 [channel (70) × frequency-band (6)]. The features are normalized using z-score normalization (Kochendörffer, 1965) before proceeding to the feature selection step.

To note that as the feature values are normalized, the time-shift-related phase offset does not show up in the normalized PPA features. Here, the normalization confines the values of PPA features to ± 1 rad. Therefore, the normalization reduces the arbitrariness of the range of the computed PPA values which is a standard step to feed extracted fetaures for modelling. Accordingly, it also confines the time-shift of the data (phase shift of the features) to the same range.

### Feature Selection

**Fig. 2:**
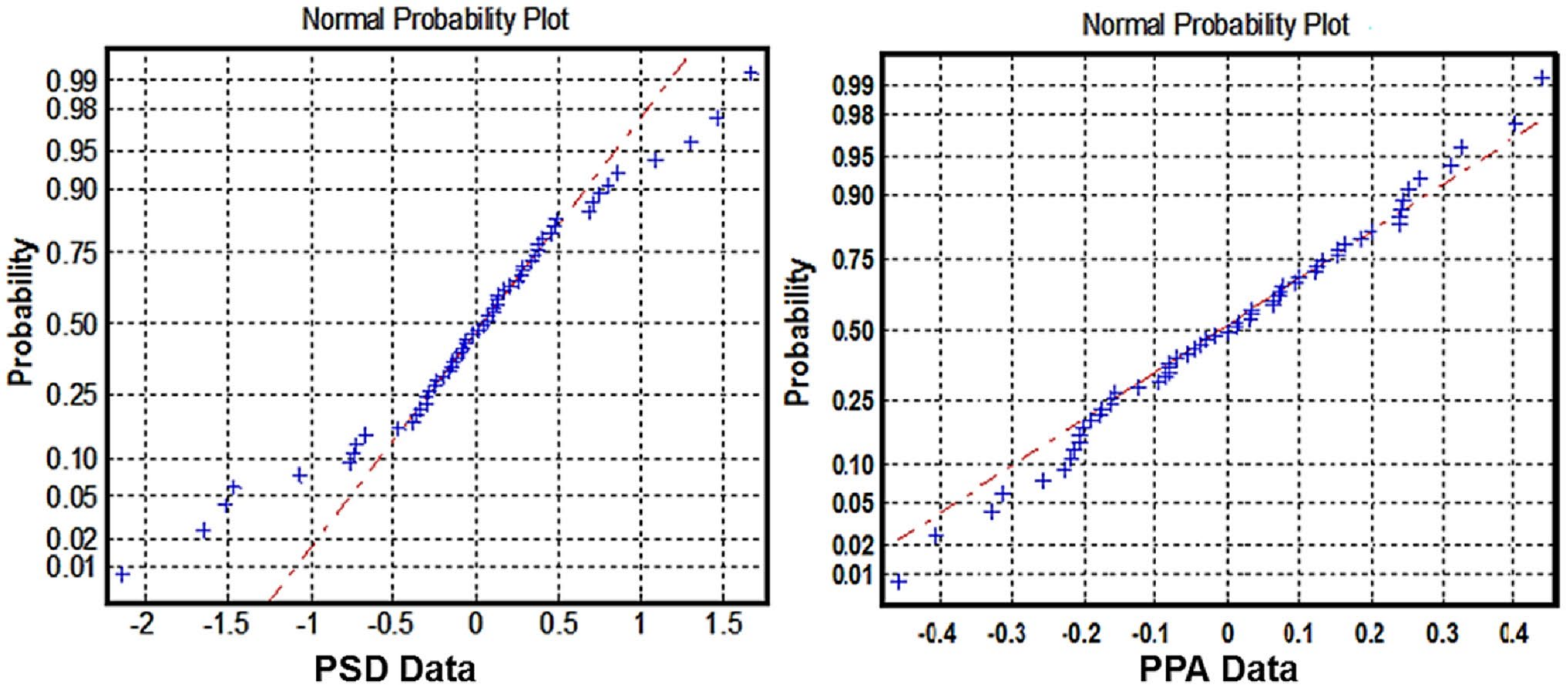
Representation of normality plot of PSD and PPA data averaged for lower gamma band features (randomly chosen frequency band)

The objective of feature selection is to extract a subset of the most relevant features while removing redundant ones (Yu & Liu, 2003). Feature selection is helpful as it reduces feature dimensions. When there are many features, the simple filter feature selection method, such as *t*-test, performs better than the complex wrapper and embedded techniques (Haury et al., 2011). We used a *t*-test and ensured the normality assumption (see Fig. 2). Even it is to be noticed that the relevance ranking strategies (e.g., *t*-test) take moderately less calculation time (Chandrashekar & Sahin, 2014) for feature selection. We have used *p*-values to rank the features. The *p*-value is the probability of obtaining the calculated *t*-statistics. From the ranked features, we selected a subset of the features that were below the chosen *p*-value. These *p*-values are used as *thresholds* to obtain a coarse selection of features to reduce the feature dimension. Different thresholds were employed to investigate the effect of increasing the number of selected features (Mwangi et al., 2014; Wang et al., 2014).

### Classification Modeling

**Fig. 3:**
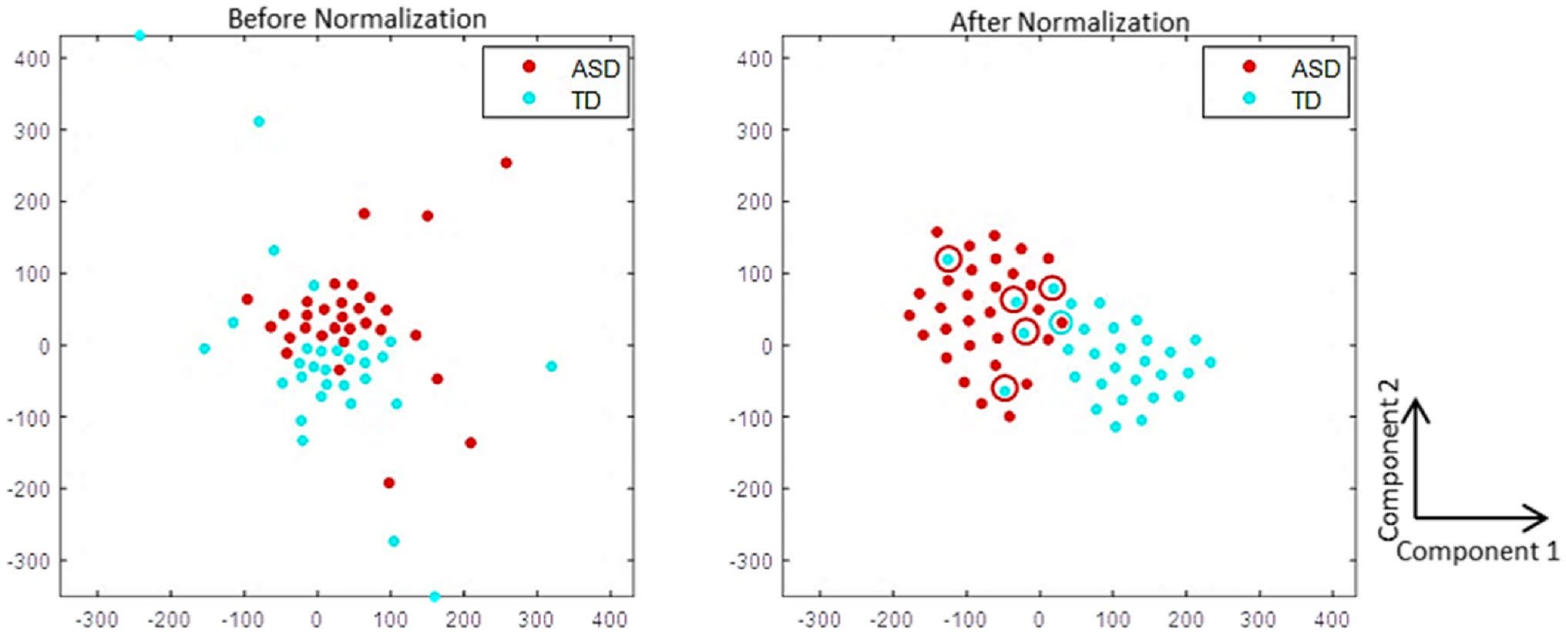
Representation of the t-SNE plots before and after normalization (over all the discriminative PPA features) of ASD and TD class

After feature selection step, the classification model finds the underlying associations or relationships among the normalized features to arrive at the decision model. Using t-distributed Stochastic Neighbor Embedding (t-SNE) plots (Hinton & Roweis, 2002), which convert high-dimensional data into its two-dimensional analogues, we present a visualization of the clustering effect for the fetaures extracted from TD and ASD classes in Fig. 3. This t-SNE embeds high-dimensional points in low dimensions solely based on their relative similarities that correspond closely to the true labels. Figure 3, also shows reduction in the number of outliers after z-score normalization which helps in modeling by making the decision boundary simpler, thereby, enhancing the classification model's discriminative ability.

**Fig. 4:**
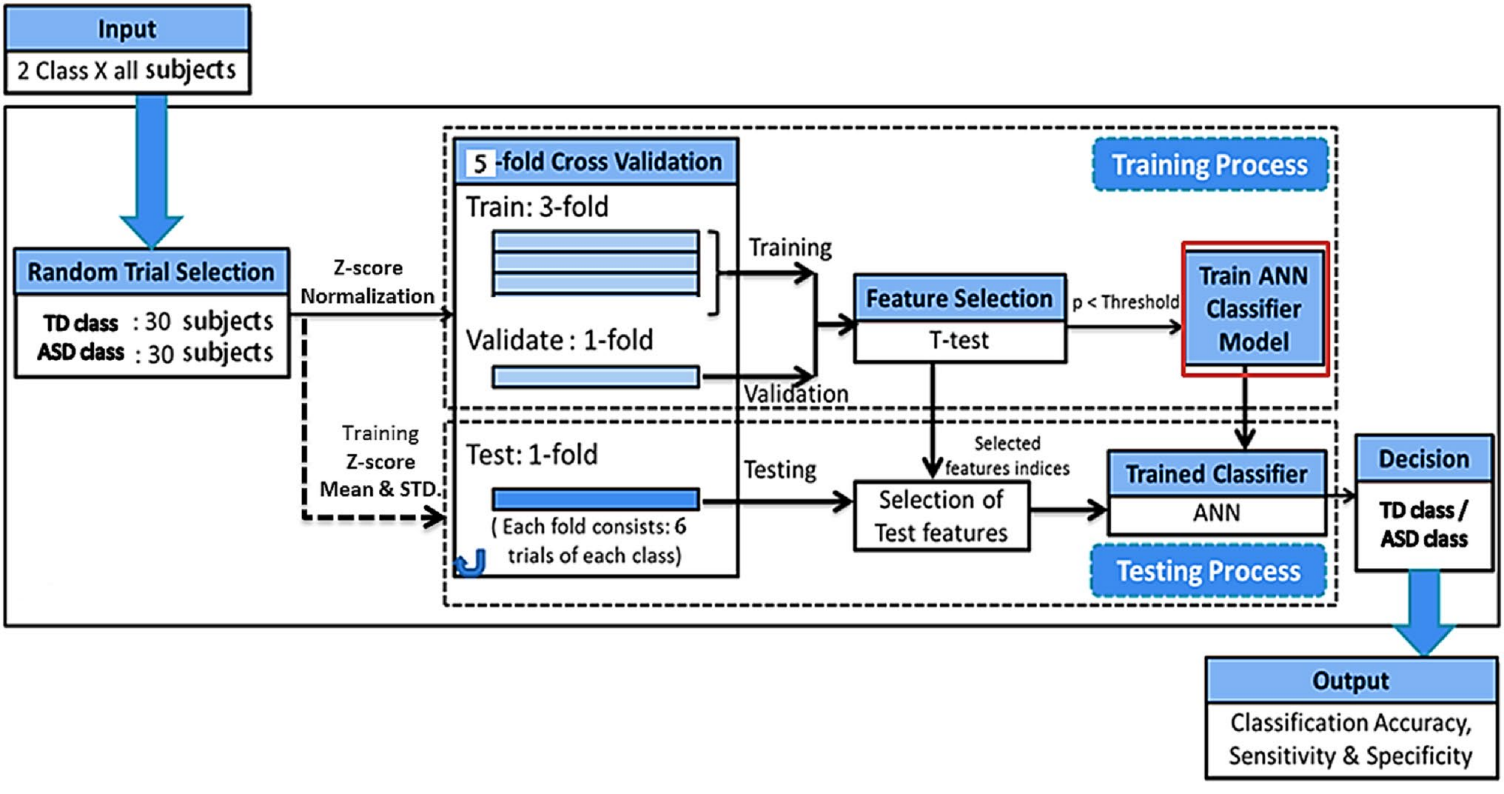
Block diagram of classification process: All subjects proceeded to the main classification block. Then typical machine learning classification process is executed with a fivefold nested cross-validation technique. Here simple filter feature selection technique (*t*-test) is followed by Artificial Neural Network for the two-class problem. Finally, the outcomes are classification accuracy, sensitivity, and specificity

This work aims to study and analyze how the underlying neural mechanisms of autistic children differ from typically developing children based on a machine learning framework. Here, MEG signals are used to classify ASD children from TD children. The detailed classification process through a block diagram is illustrated in Fig. 4. It must be stressed here that the training data set and test data are completely independent in terms of different subjects, i.e., the subjects of the test dataset are different from the subjects of the training dataset. The problem of distinguishing between the TD and the ASD children is posed as a two-class classification problem where the classification accuracy indicates the distinguishable ability of the spectral features. In this framework, a two-layered feedforward back-propagation artificial neural network (ANN) (Bishop, 1995; Haykin & Network, 2004) is used as a classifier. This ANN model finds the underlying relationships among these features for a particular class or category by learning the training data. The ANN is trained through an iterative, backpropagation algorithm which is essentially nonlinear and finds higher order statistical distribution related patterns or relationship embedded in the normalized feature vectors. This underlying relationship comes from the relative positioning of the feature vectors that remains invariant for a particular class and caters to the decision making process. We incorporated fivefold cross-validation (CV) in our classification model to remove the confusion of data biases. In this ANN model, a hidden layer consisted of 10 neurons, and an output layer included two neurons indicating the two classes. The scaled conjugate gradient descent algorithm (Møller, 1993) is used to train the neural network, as it converges fast and does not get stuck on local minima.

For the analysis using ANN, the hyperbolic tangent sigmoid transfer function is set as activation function, and the analysis has been carried out considering 10,000 as an upper limit for cycles and 10e-5 as mean square error. Normalization of feature vector is embedded before classification, and this z-score normalization rescales the feature values such that the mean of all of the values is 0 and the standard deviation is 1 (Kochendörffer, 1965). Early stopping criteria are employed in the validation set to restrict the overfitting of the current model. In this classification process, the three subsets are training, validation, and testing. The available data of all participants are divided into three subsets, with test set subjects differing in each fold of the fivefold CV. The following is the distribution of all 60 patients in each fold: the training set has 36 participants (18 from each class), the validation set contains 12 subjects (6 from each class), and the test set contains the remaining 12 individuals.The first subset is a training set, wherein gradient computation, network weights, and bias updation are performed. The second subset (validation set) validates the errors throughout the training process. Our model tried to minimize the errors through the validation process from the beginning. The continuation of this process is stopped when the validation error showed reversal trends. Here, the stopping criterion is the maximum validation error increasing check up to 6 times. Finally, the model with minimal validation error is used for the testing process. The third subset, i.e., the test set, evaluates the average classification performances over all CV. The fivefold CV technique is used with mutually exclusive test sets to calculate the performance matrices in our classification process. Our model calculated the classification accuracy, sensitivity, specificity, and standard error of the mean (SEM) for all the feature types. Here, sensitivity and specificity referred to the percentages of ASD and TD children correctly identified, respectively. Finally, the percentage to correctly classify the ASD and TD children in their respective classes is indicated as the classification accuracy. In this experiment, we calculated average classification accuracy, which averaged over fivefold classification outcomes.

### Fusion

It is challenging for any pattern classification task to model the perfect one by only one hypothesis. An effective fusion method is necessary to combine information from multiple single modality systems in a multimodal experimental framework. Information from multiple sources of different attributes can be consolidated at several levels, including the feature extraction level, score level, and decision level. This paper examined the feature level's performance and the sum rule-based score level fusion.

#### Feature-Level Fusion

All the features are concatenated to form a single feature vector (Ross et al., 2005). The final fused vector is obtained by simple concatenation of normalized feature vectors of frequency band-wise power spectrum density (*PSD*_*fb*_), and frequency band-wise preferred phase angle (*PPA*_*fb*_) features into a single vector (Nadheen & Poornima, 2013). Let *t*_*i*_ = *{*$t_{1} ,t_{2} ,t_{3} , \ldots t_{n}$*}* and *s*_*i*_ = *{*$s_{1} ,s_{2} ,s_{3} , \ldots s_{n}$*}* be the normalized feature vectors of PSD and PPA, respectively. The fused vector after feature level fusion (${\varvec{fv}}_{FeatureLevel}$) is represented as

4 $${\varvec{fv}}_{FeatureLevel}\,=\,\left\{ {t_{1} ,t_{2} ,t_{3} , \ldots t_{n} ,s_{1} ,s_{2} ,s_{3} , \ldots s_{n} } \right\}$$

The PSD and PPA features of individual subjects are obtained in the testing phase and then preprocessed to extract their feature vectors. Finally, the feature vectors are fused to form the final test feature vector. The feature-level fusion results in large feature dimensions, and the curse of dimensionality are mitigated by feature selection.

#### Score-Level Fusion

In score-level fusion, the classifier output is combined such that appropriate weights are given to the decisions of different participating systems. Score-level fusion is commonly preferred in multimodal biometric systems because matching scores contain sufficient information to make genuine and impostor cases distinguishable, and they are relatively easy to obtain (He et al., 2010). First, the matching probability scores of the individual feature types are found at the output of the classifier model. Then, the transformed scores are combined using one of the integration methods. There are different well-known methods of score-level fusion, namely, a simple sum of scores, product rule, maximum score, minimum score, and weighted sum rule-based fusion (Varchol et al., 2008). In our study, we obtained a weighted summation of the output scores according to:

5 $${\varvec{fv}}_{ScoreLevel}\,=\,\mathop \sum \limits_{i = 1}^{M} w_{i } P\left( {s_{i} } \right)$$

where $${\varvec{fv}}_{ScoreLevel}$$ is the fused vector after score-level fusion. Here, $$s_{i}$$ of Eq. (5) represents an *i*^*th*^ model of the multimodal system. The $$P\left( {s_{i} } \right)$$ is represented as the posterior probability score from the model $$s_{i}$$., and *(i* = *1,…,M)*, where *M* is the number of modalities. Weights ($$w_{i} )$$ are assigned to individual modalities. We empirically gave weights to the classifiers’ probabilistic outputs, and the values of empirical weights (*w*_*k*_*)* are chosen from 0.1 to 0.9 (where *k* = 1: 9). Here PSD and PPA are the only two models (M = 2) available, with $$P\left( {\mathbf{t}} \right)$$ and $$P\left( {\mathbf{s}} \right)$$ being the probability matrices, respectively.

6 $${\varvec{fv}}_{ScoreLevel} = w_{k } P\left( {\mathbf{t}} \right) + \left( {1 - w_{k } } \right)P\left( {\mathbf{s}} \right)$$

In our case, the system wh better performance should be given more weight in the decision-making process. This aspect makes score-level fusion superior to feature-level fusion. Here weights are chosen empirically (Waldekar & Saha, 2018) as it took less computation time than multimodal weight optimization algorithm.

#### Criteria for Fusion

**Fig. 5:**
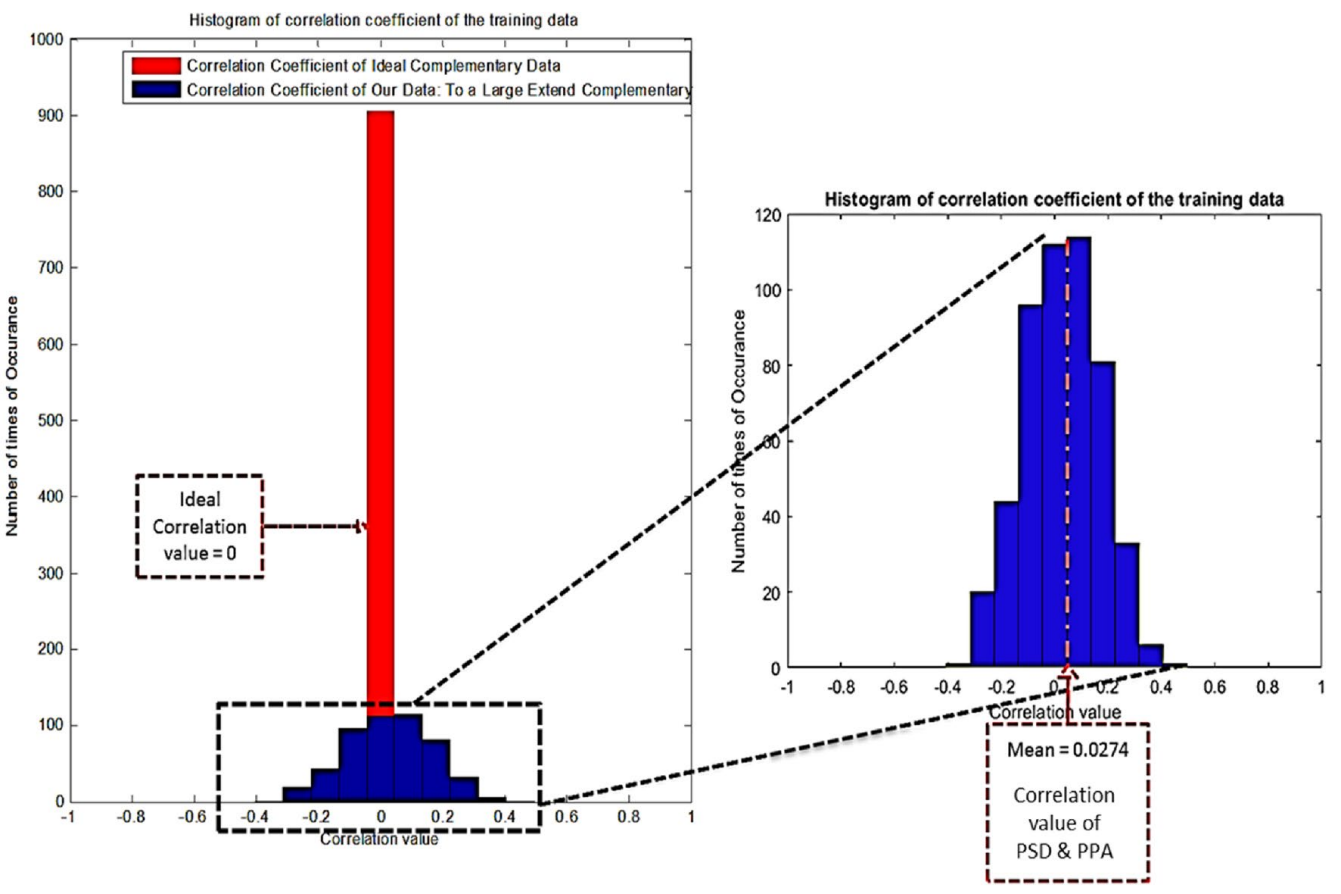
Histogram of the correlation coefficient of the training scores of PSD feature and PPA feature. The histograms are near to correlation value 0, i.e., our features are to a large extent complementary. Histogram of correlation coefficient of the training scores of two ideal complementary feature types (in ‘red’ color) and the histogram of the correlation coefficient of the training scores of PSD feature and PPA feature (in ‘blue’ color). Blue histograms are nearer to correlation value 0, i.e., our features are to a large extent complementary (mean correlation value = 0.0274)

Though theoretically, power and phase are independent of each other, we have to check whether fusion can be applicable in these (PSD and PPA) feature sets before implementing a fusion-based model. For one sample, if the classification score of one feature is different from that of the other, it implies that the two features are complementary. In other words, if two features are complementary, the correlation coefficient of their scores is expected to be close to 0. Figure 5 represents the histogram of the correlation coefficient of the training scores of the PSD feature and PPA feature. If the two feature types each have *n* feature vectors, we will get *n-*correlation coefficients. It is necessary to check the mean of these *n-*correlation coefficient values spread out across a range of values near to 0. Here the mean correlation coefficient value is 0.0274, indicating that the PSD and PPA features are complementary. As the correlation coefficient between the scores of two features is projected to be close to zero, the two feature types (PSD and PPA) are complementary to some extent.

## Results

### PPA and PSD Based Classification

The analysis of $PPA_{all}$, $PPA_{Left}$ and $PPA_{Right}$ features were carried out for each participant. To compare the performances with PSD features, we have investigated $PSD_{all}$, $PSD_{Left}$ and $PSD_{Right}$ features. $PPA_{all}$ and $PSD_{all}$ features were chosen from all 151 MEG sensors. The $PPA_{Left}$ and $PPA_{Right }$ correspond to the left and right hemispheric sensors, respectively. Hemispheric features were similarly considered for $PSD_{Left}$ and $PSD_{Right}$ features. In these hemispheric features, 11 midline sensors were excluded. Hence, in each hemisphere, there are 70 sensors only.

**Fig. 6:**
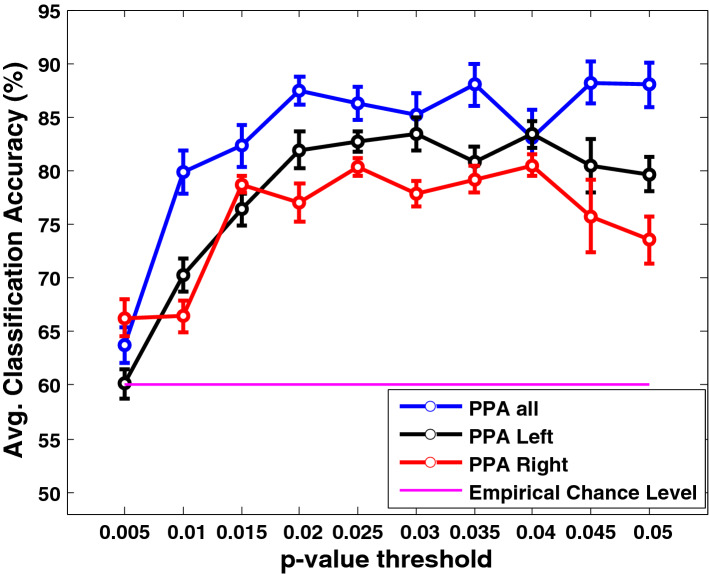
Representation of average classification accuracy (in %) of different preferred phase angle (PPA) features (for the whole brain and each hemisphere) with respect to each *p*-value threshold (from 0.005 to 0.05) along with empirical chance level (pink horizontal line)

The classification outcome of PPA features illustrated in Fig. 6. The classification accuracy was graphically illustrated with the empirical chance level of 60% (Combrisson & Jerbi, 2015) by setting the *p*-value threshold in between 0.005 to 0.05 with the interval of *p*-value 0.005. Thus, a suitable threshold was empirically ascertained for selecting the features. With the stricter *p*-value threshold, selecting discriminating features becomes less, resulting in degradation in the classification accuracy. As the classification accuracy did not show substantial improvement than the chance level, the *p*-value was gradually increased to find out the optimal threshold beyond which the classification accuracy almost saturated. The optimal *p*-value was chosen using the classification results of the validation set data only.

Table 1 shows the classification performances of the ANN classifier using these PPA feature types, whereas similarly, Table 2 represents the same for PSD feature types. We only mentioned the decision outcomes at optimal *p*-values for each feature type. Comparing the results of both the features, it is found that PPA features yield better classification accuracy than PSD features for ASD classification in children from their resting state MEG data. The classification performance of *PPA*_*all*_ is 88.20 ± 3.87%, whereas the same for *PSD*_*all*_ is 82.13 ± 2.11%. For PSD features, right hemispheric features contained more discriminative information than the left hemispheric ones. However, for PPA features based on classification outcome, we have found that the distinguishing information of either left or right hemisphere was comparable. The hemispheric differences were statistically tested for both the PPA and PSD features. The statistical *t-*test indicated that the hemispheric PPA feature groups (i.e.,$PPA_{Left}$ and $PPA_{Right}$) did not significantly differ in performances (*p* > 0.95). However, the hemispheric PSD feature groups significantly differed (*p* < 0.002) in fold-wise test accuracy, according to a paired *t*-test.

**Table 1:** Representation of classification outcomes (in %) of PPA features of overall cortex along with left and right hemisphere

Feature: PPA	PPA_all_	PPA_Left_	PPA_Right_
*p-*value <	0.045	0.040	0.040
Accuracy ± SEM (%)	88.20 ± 3.87	83.40 ± 2.48	80.47 ± 2.01
Sensitivity ± SEM (%)	90.80 ± 3.24	86.53 ± 2.62	80.93 ± 1.71
Specificity ± SEM (%)	85.60 ± 5.61	80.26 ± 3.56	80.00 ± 3.52
Number of selected features	60	23	29

**Table 2:** Representation of classification outcomes (in %) of PSD features of overall cortex along with left and right hemisphere

Feature: PSD	PSD_all_	PSD_Left_	PSD_Right_
*p-*value <	0.015	0.015	0.015
Accuracy ± SEM (%)	82.13 ± 2.11	73.93 ± 2.53	80.87 ± 4.68
Sensitivity ± SEM (%)	82.13 ± 3.62	74.53 ± 6.90	77.20 ± 7.93
Specificity ± SEM (%)	82.13 ± 2.46	73.33 ± 4.96	84.53 ± 3.02
Number of selected features	46	15	26

**Fig. 7:**
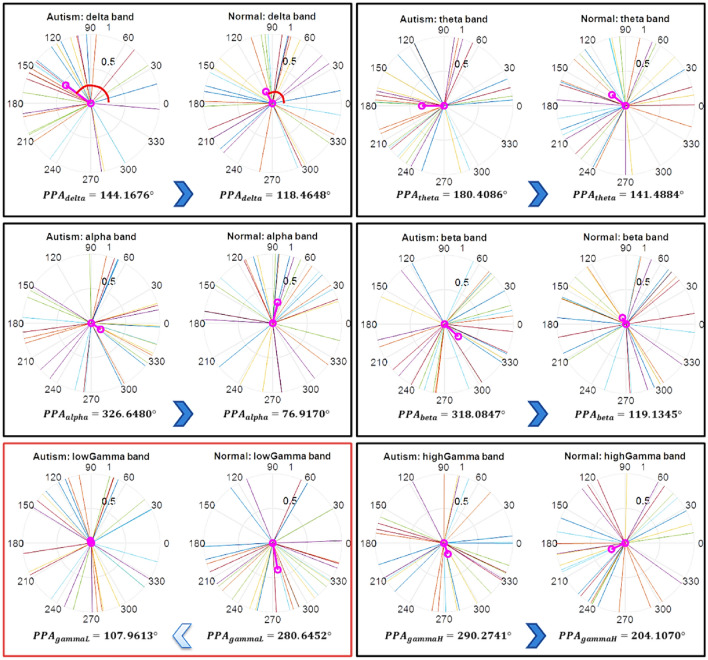
Representation of frequency band-wise preferred phase angle range in both ASD class and TD class and the mean angle of that PPA features (red color) in each class at the randomly chosen AG102 sensor (located at right parietal area) before normalization. The mean angle of PPA of all 30 subjects (angle of the magenta line) reflects that the mean angle of PPA of the ASD class is greater than the mean angle of the PPA of the TD class in all frequency bands except the low gamma band

By comparing the results of Tables 1 and 2, this phase based feature (*PPA*_*all*_*)* demonstrated superior (88%) classification performance over the one (82%) based on commonly used power spectral density (*PSD*_*all*_) based feature. As a consequence, this phase-based feature is investigated further. The mean angle of the PPA features of each frequency band is illustrated here in polar plots of Fig. 7 for both ASD and TD classes. It represents frequency band-wise PPA features at the AG102 sensor (located at right parietal area) for 30 participants before normalization. To give an example, we randomly chose one sensor (AG102) for each of the frequency bands. Observations from this Fig. 7 reflect that the PPA value ranges from 0° to 360°. The mean angle of PPA (angle of the magenta line) reflects that the mean angle of PPA of the ASD class is greater than the mean angle of the PPA of the TD class in all frequency bands except the low gamma band.

**Fig. 8:**
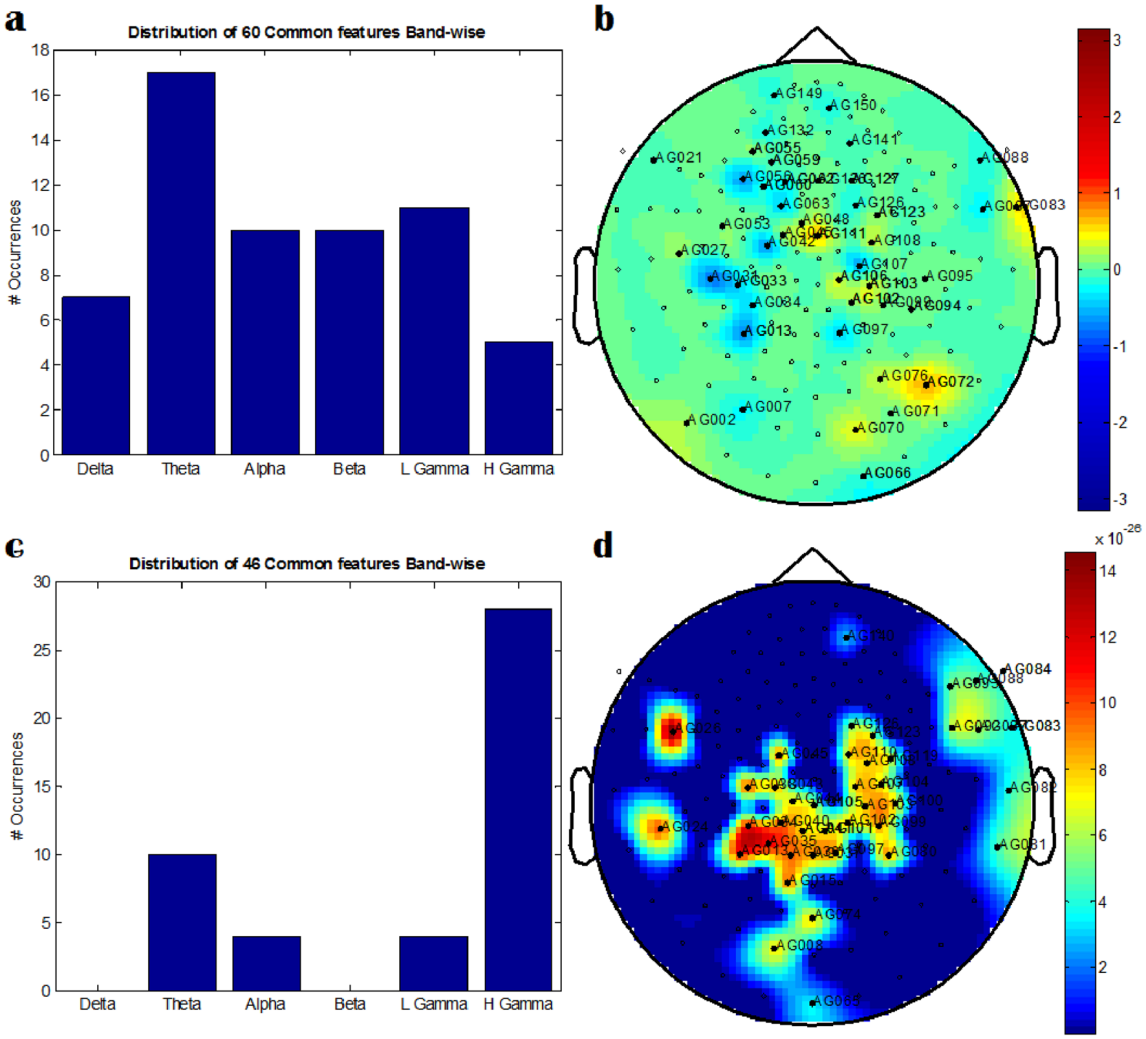
Representation of discriminating features: (**a**) the frequency band-wise distribution of selected PPA features from all 151 channels (*PPA*_*all*_). (**b**) Topoplot represents the spatial pattern distribution of discriminating *PPA*_*all*_ features. The topoplot color scales indicates PPA, ranges from − pi to + pi (from − 3.141 to + 3.141) radians. The distribution of 60 discriminating sensors in the brain and their corresponding PPA values are represented in the layout. (**c**) Similarly, the frequency band-wise distribution of selected *PSD*_*all*_ features. (**d**) The spatial pattern distribution of discriminating *PSD*_*all*_ features is represented in this topoplot. The distribution of 46 sensors in the brain and their corresponding brain signals are represented in the layout. The color scale is 10^−26^ *[− 0.14 to + 14.41] T^2^ (or 1.0e−28 *[− 0.00143 to 0.14417] Tesla^2^). The value represents the brain signal amplitude square at those particular sensors averaged over all the 3 second data

Using a data-driven approach, we can classify autistic children from normal children. However, this is not the only target of this study. We also explored neural oscillations and spatial pattern analysis to identify the frequency bands and spatial patterns that consistently show discriminative ability between two classes across all the subjects. Most discriminating PPA features are found here from the theta frequency band. Spatial patterns of PPA discriminatory features are located in the central, parietal, and frontal brain regions. However, in this frequency band-wise spatial pattern analysis, discriminating PSD features are found mostly from high gamma-band oscillations, and these discriminating features are mostly selected from central, parietal, and temporal regions. The row-wise representation of Fig. 8 represents the discriminatory features of *PPA*_*all*_ and *PSD*_*all*_. The frequency band-wise distribution of selected features is illustrated in Fig. 8a,c and the spatial pattern of those features ispresented in topoplots of Fig. 8b,d.

### Fusion-Based Classification

**Fig. 9:**
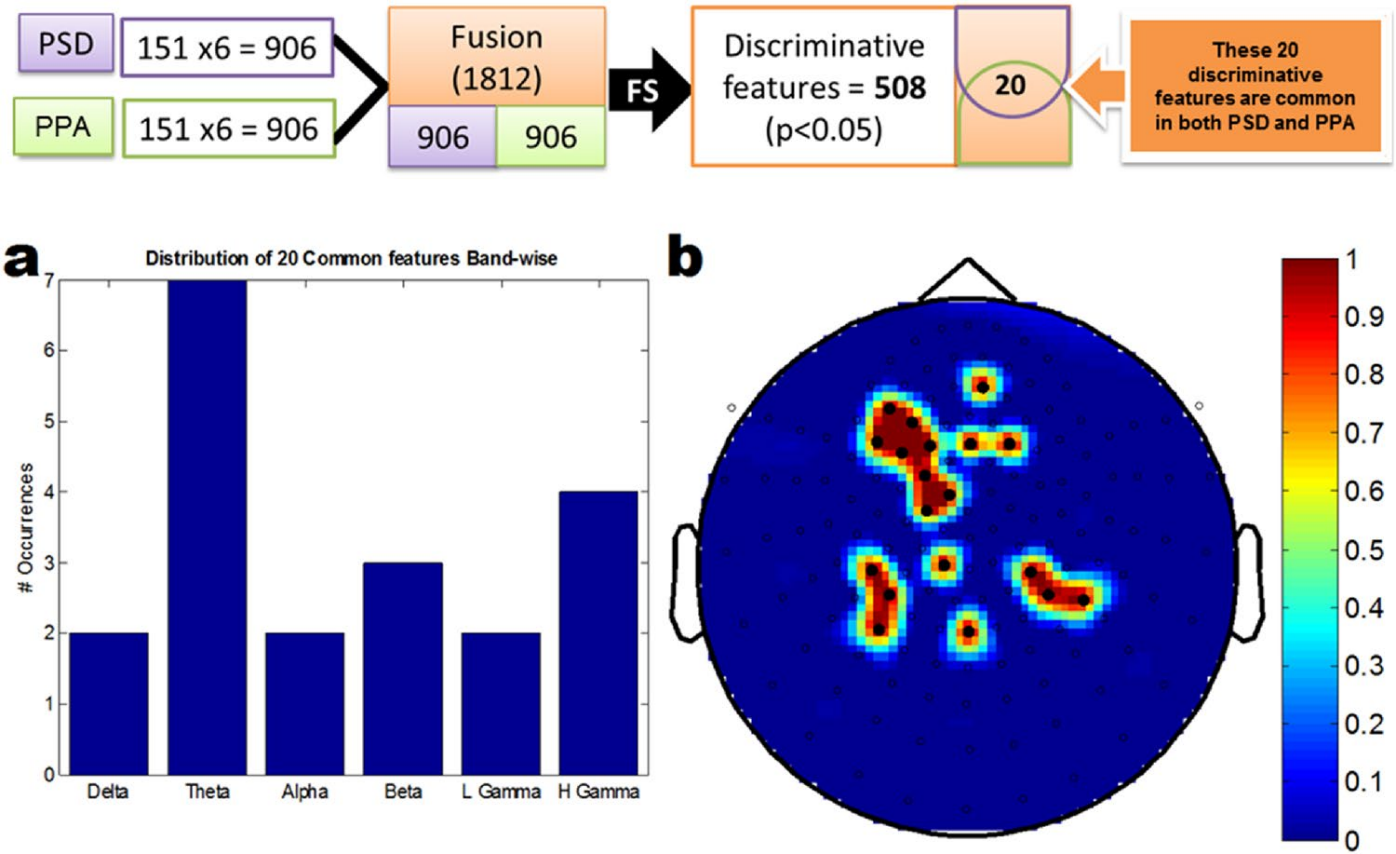
Representation of discriminating features of the fusion based system: (**a**) the frequency band-wise distribution of selected 20 features, common in both PSD and PPA feature types. (**b**) Topoplot represents the spatial pattern distribution of these discriminating features. The topoplot color indicates "count"

For feature-level fusion, we combined *PSD*_*all*_ and *PPA*_*all*_ features for each participant. Since the feature dimension was large, we performed feature selection as mentioned earlier. Following ANN based classification, we only choose the optimal *p*-value, i.e., *p* < 0.05 (the highest threshold) for both the PSD and PPA features. After feature-level fusion, the average classification accuracy of features is 94.46 ± 1.35% for the specific *p*-value mentioned above. The sensitivity, specificity, and the number of selected features are 94.13 ± 2.90%, 94.80 ± 2.52%, and 508, respectively. Among these 508 discriminative features, 20 discriminative features are common in both PSD and PPA. These discriminative features are mostly from theta-band oscillations, which correlate with autistic symptomatology. The discriminating features, those are common in individual feature types, are shown in Fig. 9. Notably, the feature-level fusion of PSD and PPA features performance is, on average, 6% better than the classifier based on PPA features.

For score level-fusion, the classification performance of sum-rule based score-level fusion is 98.33 ± 0.74% at *p-*values threshold on < 0.02. Here probability weight is set to 0.2 (*w*_*k*_) of PSD features, and that for PPA feature is 0.8. Table 3 shows the classification performances of ANN classifier using these fusion-based models, i.e., feature-level fusion and score-level fusion types. We only mentioned the decision outcomes at optimal *p*-values for each fusion-based model type. Using ANN, the performance of score-level fusion has improved 4% compared to feature-level fusion.

**Table 3:** Representation of classification performances (in %) of fusion based models of overall cortex

Fusion	Feature-level fusion	Score-level fusion
*p-*value <	0.05	0.02
Accuracy ± SEM (%)	94.46 ± 1.35	98.33 ± 0.74
Sensitivity ± SEM (%)	94.13 ± 2.90	98.70 ± 0.52
Specificity ± SEM (%)	94.80 ± 2.52	97.96 ± 1.49
Number of selected features	506	86 (PPA:31; PSD:55)

**Fig. 10:**
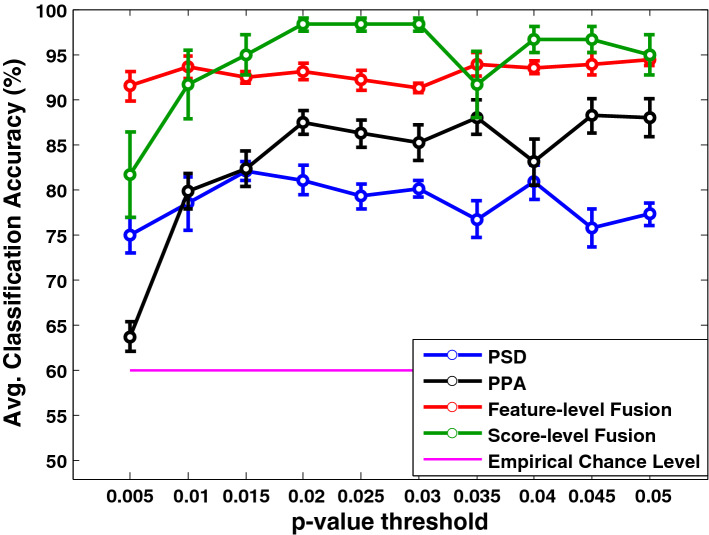
Representation of average classification accuracy of *PSD*_*all*_, *PPA*_*all,*_ and fused feature vector after feature-level fusion and score-level fusion with respect to different *p*-value thresholds used in the feature selection method. The average accuracy is presented with the empirical chance level (pink horizontal line). Error bars indicate the standard error of the mean (SEM)

**Fig. 11:**
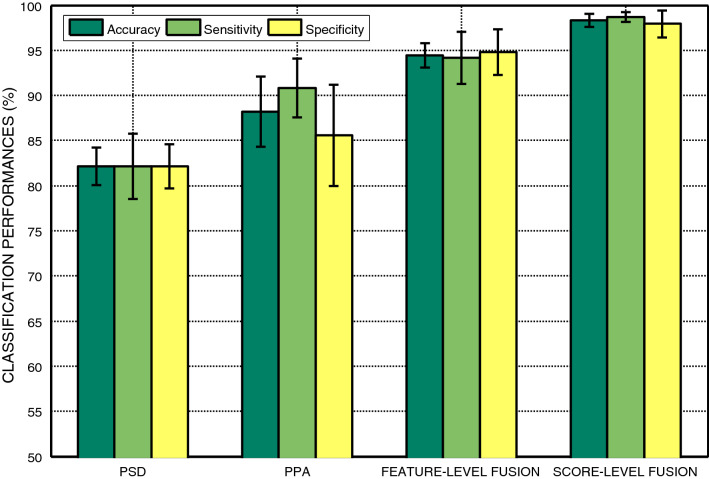
Representation of classification performances (in %) of individual features, feature-level fusion, and score-level fusion

Figure 10 shows the average classification performance of power spectral density (*PSD*_*all*_) features, preferred phase angle (*PPA*_*all*_) features, and fused feature vector after feature-level fusion and score-level fusion concerning different *p*-value thresholds used in the feature selection method. After feature and score-level fusion, using spectral-domain analysis, we achieve a classification accuracy of 94% and 98%, respectively, thereby score-level fusion offering a slightly better classification accuracy than the feature-level fusion. Finally, we show the comparison of classification accuracy, sensitivity, and specificity at individual feature level (PSD and PPA), feature-level fusion, and score-level fusion in Fig. 11 through barplot.

**Fig. 12:**
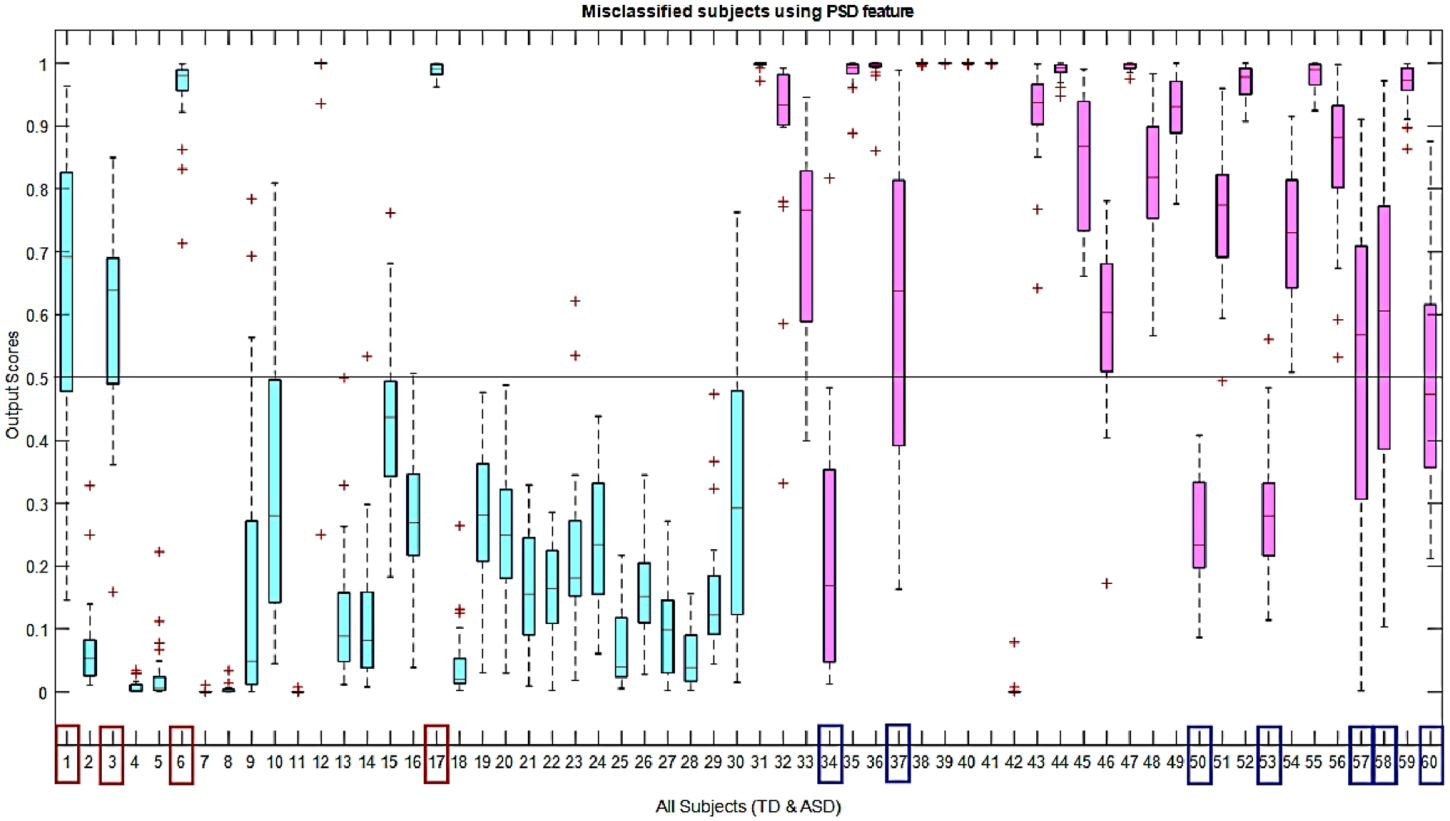
Representation of classification output scores of PSD features with respect to all 60 subjects. PSD misclassified 11 subjects and the subject indices are: 1, 3, 6, 17, 34, 37, 50, 53, 57, 58, 60. The first 30 boxplots (in ‘blue’ color) represent TD class and rest 30 represent ASD class

**Fig. 13:**
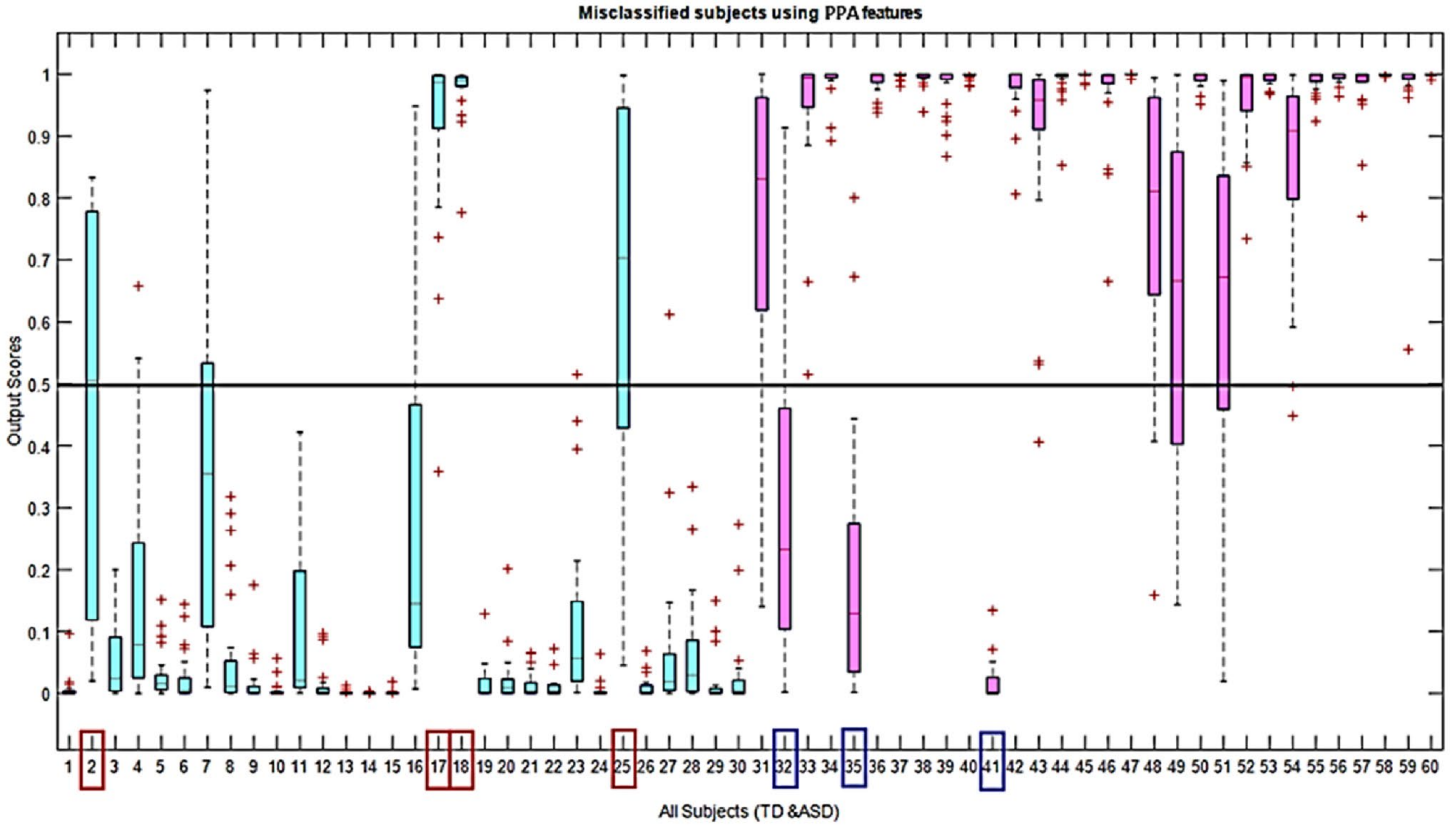
Representation of classification output scores of PPA features with respect to all 60 subjects. PPA misclassified 7 subjects and the subject indices are: 2, 17, 18, 25, 32, 35, 41. The first 30 boxplots (in ‘blue’ color) represent the TD class, and the rest 30 represent the ASD class

In autism detection using MEG signals, this fusion based model is a novel machine learning approach. To understand how efficient this fusion of PSD and PPA features is, we must check which subjects are misclassified in individual models. It is illustrated using boxplots in Figs. 12 and 13 that the PSD model misclassified 11 subjects and PPA misclassified 7 subjects, respectively. The first 30 boxplots (in ‘blue’color) represent the TD class, and the rest 30 represent the ASD class. The TD subjects are correctly classified if the output scores are ≤ 0.5, and ASD subjects are correctly classified if those scores are ≥ 0*.*5. Among these sets of subjects, only the 17th subject is commonly misclassified. Except for this (17th) subject, different sets of subjects are misclassified from the ANN model, using each model (with PSD and PPA features). Hence, from the perspective of classification scores or output, it is evident that the PSD feature cannot misclassify a different set of subjects than the PPA characteristics. Instead of criteria of fusion of individual features, here on the other way again, we showed that two individual models are complementary; hence multimodal fusion is more effective to classify ASD and TD in this machine learning framework.

## Discussion

In this study, we compared the ongoing neuromagnetic brain activity of young children (4–7 years) with autism spectrum disorder (ASD) to age and gender-matched typically developing (TD) children. Using features based on spectral-domain analysis (both in power and phase) along with the fusion analysis in a machine learning classifier, we exhibited that we could classify the ASD from TD children with an accuracy well above chance level. Further, we found that the preferred phase angle (PPA) showed higher classification accuracy than the traditional power spectral density (PSD) feature. However, by fusing these two features, we demonstrated a considerable improvement of classification accuracy, thereby suggesting the complementarity of these two features in distinguishing ASD from TD children. In this paper, using spectral features, we have proposed a fusion-based classifier model with a supervised classification method. However, in clinical practice, in new and realistic conditions, we will get the MEG signals of other new sets of children with their screening tool test scores which will indicate their class labels. Therefore, we can test the spectral features extracted from the MEG signal of a new set of children using our experimental protocol and considering our model as a trained model. Here, the classification accuracy reported in this paper reflects how accurate our classifier is and how proficient this experimental method is to distinguish autistic children from TD children using their MEG signal. In the following paragraphs, we discuss each of these findings in further detail and include some limitations of the current study.

### Preferred Phase Angle in Autism

We employed a pattern classification approach using artificial neural network (ANN) based modelling for discriminating ASD and TD children using PPA features. As noted that phase angles are independent of power (Cohen, 2014), we compared our results with power spectrum density (PSD) features that capture the mean amplitude of the neural oscillations in each frequency band. We found that PPA yields better classification accuracy than PSD, suggesting that phase angles aggregated in each frequency reveal significant phase alignment effects that distinguish autistic brain function from typically developing brain. Furthermore, in spectral analysis, the phase-based measures reflect considerable improvement over power-based measures, giving insight into the presence /absence of systematic rhythms across oscillations in each neural frequency band. Hence, this result suggests a new way to quantify temporal synchronization of brain oscillations as an effective measure in discriminating ASD from TD children.

According to the E-I hypothesis, when the neuronal membrane potential is balanced, the relative phase and magnitude of excitation-inhibition (E-I) are also balanced, reflecting systematic synchronous neuronal oscillations in the brain (Sohal & Rubenstein, 2019; Zhou & Yu, 2018). The imbalance of E-I can disturb this signature of relative neural firing time and magnitude, reflecting synchronous neural oscillation’ power and phase. Hence, spectral power and phase are disrupted in functioning. Our proposed feature PPA measures the resultant phase angle of frequency bins over a frequency band. It reflects the phase of excitability of the underlying neuronal assemblies oscillating at different nearby frequencies but belonging to a frequency band. It is not directly linked to the phase of the membrane potential. Instead, PPA may be linked to the cortex’s imbalanced synaptic excitation and inhibition during the resting state and sensory processing. Our experimental outcome shows that the phase of excitability of underlying neurons of the autistic brain is different from the typically developing brain in each frequency band.

### Frequency Band-Wise Preferred Phase Angle in Each Class

The length of the average phase angle vector in polar space within a typical frequency band is known as phase consistency (Cohen, 2014), and the angle of this resultant vector is known as the preferred phase angle. Phase angles are commonly represented in polar space, ranging from 0 to 2*π*. These can be illustrated as vectors on a unit circle. These PPA ranges are different in each class, particularly for discriminant features. Frequency band-wise observations in phase based feature reflect that the PPA of ASD class is greater than PPA of TD class in all frequency bands except low gamma (Fig. 7). PPA represents how much phase synchronization within a frequency band deviates from the initial phase in a counter-clockwise direction. Using PPA, it is clear that the initial phase angle deviation of phase in autism class is more than in TD class.

### Neural Oscillations and Spatial Pattern Analysis

Using a data-driven exploratory approach, we showed that it is possible to classify ASD from TD children. However, we also explored the ASD pathophysiology by performing frequency band-wise analysis and spatial pattern analysis of the features over whole cortex MEG sensors. More speculatively, the results of neural oscillations and spatial pattern analysis are presented in Fig. 8. In the case of PPA features, neural oscillations that distinguished the ASD class from the TD class were not specific to any particular frequency band. Instead, the discriminatory PPA features were from all the frequency bands; a marginal dominance (*p-value* = 0.054) of theta band (4–8 Hz) was observed. In the spatial pattern analysis, the discriminatory features were dominant in the central, parietal, and frontal brain regions. In the case of PSD, discriminatory features were observed in the high-frequency gamma band. Discriminating power-based spectral features are found in central, parietal, and temporal brain regions. Already, NeuroSPECT work concluded that temporal and frontal lobe dysfunction is significantly involved in autism spectrum disorder (Goldberg et al., 1999). For both spectral features, discriminating features are common from central and parietal brain regions. The explanation of the relationship between autistic symptomatology and these brain areas is found in the literature (Courchesne et al., 1993; DeRamus et al., 2014). It was found that compared to TD, ASD children showed significantly enhanced activation in the parietal region (particularly the angular gyrus) while detecting the location of objects on visuospatial processing (DeRamus et al., 2014). Even parietal lobe abnormalities of autistic children were detected in neuroanatomy investigation (Courchesne et al., 1993), where the cerebrum was reported as the origin of this abnormality. The cerebrum region mediates many high cognitive functions; several of those (i.e., social communication, language, reasoning, planning, and organizing) are severely disrupted in the autistic brain.

Previous research has found abnormal high gamma spectral power in young ASD children (Lushchekina et al., 2012; Orekhova et al., 2007; van Diessen et al., 2015). Our study also found that the discriminating PSD features were generally from high gamma oscillations. Likewise, this finding aligns with the studies that identified unusual activation of gamma-band oscillations in ASD children (Lushchekina et al., 2012; Orekhova et al., 2007; van Diessen et al., 2015). In addition, this study demonstrates a rightward-lateralized neural oscillation in power analysis similar to previous literature (Cornew et al., 2012; Kikuchi et al., 2013). In the literature of the last decade, phase-based features were investigated in the connectivity analysis (O'Reilly et al., 2017; Velazquez et al., 2009; Ye et al., 2014), however not yet explored in spectral-domain analysis for the autism classification problem. In spectral analysis, the preferred phase angle is a novel feature, where phase features are implemented even in frequency band-wise clustering. Our proposed feature yields better classification accuracy (88.20 ± 3.87%) than the PSD features (82.13 ± 2.11%). So we suggest that PPA can also be used as a useful measure to identify early ASD in children from their ongoing brain responses.

### Analysis of Fusion Based System

To explore the frequency band-wise analysis and spatial pattern analysis of the fusion-based system, features over all 151 sensors were also investigated. The average classification performance in the feature-level fusion-based system is 94.46 ± 1.35% for the *p*-value < 0.05. In this specific *p*-value threshold (*p* < 0.05), the number of selected features is 508. Among these 508 discriminative features, 20 discriminative features are common in both PSD and PPA (Fig. 9). These discriminative features are mostly from theta-band oscillations, corresponding to autistic symptomatology. Finally, this fusion-based system estimates are correlated with ASD scores indicating clinical relevance of the feature level fusion of PPA and PSD matrices.

### Role of Theta Band in Autism

In this study, autism detection in young children is extended using spectral features based on large-scale neural oscillations in a machine learning classifier in a fused system. Overall this work demonstrates the resting-state neural function aberration, a key component of the autism symptom profile (Kessler et al., 2016). Even to distinguish ASD children from TD children, disrupted oscillatory synchronization is found in multiple frequency bands as reported in (Simon & Wallace, 2016). In the current study, theta oscillations are found as discriminative features closely linked to changes in memory state, memory performance, and auditory responses. Hence, this theta-band preferred phase angle can also be an optimal measure to discriminate ASD from TD, as autistic children have specific memory and memory strengths difficulties. Autistic children use fewer strategies spontaneously to retrieve information (e.g., visual and rote memory) (Williams et al., 2006). Multiple studies suggest that memory deficit observed in ASD has a biological explanation linked to abnormalities in the hippocampus and other neural regions responsible for strategic regulation, such as the amygdala and the frontal cortex (Wojcik et al., 2013).

### Limitations

Our approach is not without limitations. First, we restricted our analysis to the MEG sensor space only, so we cannot provide any clear conclusion about the precise involvement of underlying brain sources. Future studies could adopt advanced source localization methods that would provide a more accurate measure of brain activity to examine PPA features in ASD better. Second, both PPA and PSD features were computed at each sensor, so our findings cannot provide any direct information about the nature of information transfer between near and distant sensor regions. Third, we only analyzed a brief 3 min period of brain data, and it would be essential to obtain a longer recording to validate the robustness of our findings. Fourth, our findings suggested some complementarities between PPA and PSD; however, additional studies are needed to substantiate this claim further. For example, future studies with larger sample sizes could match children on PSD values and then determine whether PPA would still discriminate between ASD and TD (a similar approach can be adopted by first matching on PPA values). Finally, we termed our recording as resting-state brain activity while the children watched cartoons of their choice, and such natural viewing conditions might have introduced various heterogeneities in the recording. Though this type of data collection is increasingly being used in studies with young children (Richardson et al., 2018), one cannot distinguish between intrinsic and task-driven contributions to the reported patterns, both in terms of phase and amplitude, of brain oscillations. Future studies could investigate individual contributions of both task-driven and intrinsic by collecting MEG data during the task and actual resting state from the same child.

## Conclusion

The present findings demonstrate that it is feasible to differentiate autistic children from typically developing children based on their resting-state brain oscillations recorded by MEG. We have introduced a feature, preferred phase angle, which utilizes frequency band-wise phase consistency. We showed that the PPA-based classifier outperformed the PSD-based classifier, and the best classification accuracy was obtained by combining PPA and PSD in a fusion-based framework. The complementarity of power and phase-based spectral features are demonstrated in this work, where we have proposed a fusion-based machine learning framework in autism children detection. Altogether, this study suggests that characteristics of ongoing large-scale brain oscillations contribute towards the core pathophysiology of ASD.

## Data Availability

The data and codes would be made available at a reasonable request made to the corresponding author.
